# Cerebral Vasculature Influences Blast-Induced Biomechanical Responses of Human Brain Tissue

**DOI:** 10.3389/fbioe.2021.744808

**Published:** 2021-11-04

**Authors:** Dhananjay Radhakrishnan Subramaniam, Ginu Unnikrishnan, Aravind Sundaramurthy, Jose E. Rubio, Vivek Bhaskar Kote, Jaques Reifman

**Affiliations:** ^1^ Department of Defense Biotechnology High Performance Computing Software Applications Institute, Telemedicine and Advanced Technology Research Center, United States Army Medical Research and Development Command, Fort Detrick, MD, United States; ^2^ The Henry M. Jackson Foundation for the Advancement of Military Medicine, Inc., Bethesda, MD, United States

**Keywords:** blast-induced traumatic brain injury, blast overpressure, shock tube, brain biomechanical responses, finite-element model, human cerebral vasculature

## Abstract

Multiple finite-element (FE) models to predict the biomechanical responses in the human brain resulting from the interaction with blast waves have established the importance of including the brain-surface convolutions, the major cerebral veins, and using non-linear brain-tissue properties to improve model accuracy. We hypothesize that inclusion of a more detailed network of cerebral veins and arteries can further enhance the model-predicted biomechanical responses and help identify correlates of blast-induced brain injury. To more comprehensively capture the biomechanical responses of human brain tissues to blast-wave exposure, we coupled a three-dimensional (3-D) detailed-vasculature human-head FE model, previously validated for blunt impact, with a 3-D shock-tube FE model. Using the coupled model, we computed the biomechanical responses of a human head facing an incoming blast wave for blast overpressures (BOPs) equivalent to 68, 83, and 104 kPa. We validated our FE model, which includes the detailed network of cerebral veins and arteries, the gyri and the sulci, and hyper-viscoelastic brain-tissue properties, by comparing the model-predicted intracranial pressure (ICP) values with previously collected data from shock-tube experiments performed on cadaver heads. In addition, to quantify the influence of including a more comprehensive network of brain vessels, we compared the biomechanical responses of our detailed-vasculature model with those of a reduced-vasculature model and a no-vasculature model for the same blast-loading conditions. For the three BOPs, the predicted ICP values matched well with the experimental results in the frontal lobe, with peak-pressure differences of 4–11% and phase-shift differences of 9–13%. As expected, incorporating the detailed cerebral vasculature did not influence the ICP, however, it redistributed the peak brain-tissue strains by as much as 30% and yielded peak strain differences of up to 7%. When compared to existing reduced-vasculature FE models that only include the major cerebral veins, our high-fidelity model redistributed the brain-tissue strains in most of the brain, highlighting the importance of including a detailed cerebral vessel network in human-head FE models to more comprehensively account for the biomechanical responses induced by blast exposure.

## Introduction

Blast-induced injuries resulting from exposure to improvised explosive devices are a major cause of mortality and morbidity of United States Service members deployed to Iraq and Afghanistan (Hoge et al., 2008; Elder and Cristian, 2009; Ritenour et al., 2010). In fact, the Defense and Veterans Brain Injury Center estimates the incidence of blast-induced traumatic brain injury (bTBI) to be as high as 22% (Martin et al., 2008), 82% of which being mild injuries (U.S. Department of Defense, 2000-2020). Mild primary injury can result from the interaction of a blast wave with the brain (Elder and Cristian, 2009), possibly caused by stress-wave propagation through the brain (Taylor and Ford, 2009), skull flexure (Bolander et al., 2011), cavitation effects (Goeller et al., 2012), or acceleration of the head (Gullotti et al., 2014). However, given the limited availability of clinical data and uncertainty associated with the exact nature of blast exposure in examined cadavers, we do not fully understand the pathophysiology of primary bTBI.

One way to assess the effects of blast-wave exposure on brain tissues is to use computational models to predict blast-induced biomechanical responses of the brain, such as pressure, stress, and strain, which we expect to correlate with observations of brain-tissue changes and damage (Morrison et al., 2011; Meaney et al., 2014). For instance, to assess the protective features of different advanced combat helmet designs, Tan et al. (2014) and Zhang et al. (2013) independently developed three-dimensional (3-D) finite-element (FE) models of the human head and evaluated blast-induced pressures, stresses, and strains for different blast overpressure (BOP) exposures. In separate studies, Sharma (2011), Singh et al. (2014), and Garimella et al. (2018) observed a good agreement between the measured brain-tissue pressures obtained from cadaver experiments and model-predicted frontal-lobe values for low- and medium-intensity BOPs. However, these developed human-head FE models vary greatly in terms of the number of anatomical features represented, material properties of the brain tissue, brain anatomy, and description of the cerebral vasculature (Taylor and Ford, 2009; Chafi et al., 2010; Nyein et al., 2010; Sharma, 2011; Panzer et al., 2012; Singh et al., 2014; Wang et al., 2014; Cotton et al., 2016; Salimi Jazi et al., 2016; Rodríguez-Millán et al., 2017; Tan et al., 2017; Garimella et al., 2018). For example, Rodríguez-Millán et al. (2017) included 13 anatomical features in their FE model, whereas the model developed by Chafi et al. (2010) contained only eight. Although the model developed by Rodríguez-Millán et al. contributed toward improving the anatomical description of the human head, they used linear viscoelastic material properties of the brain tissues, which could possibly limit the accuracy of the model-predicted brain-tissue strains (de Rooij and Kuhl, 2016). In contrast, Sarvghad-Moghaddam et al. (2017), who used hyper-viscoelastic brain-tissue properties in their FE model to more precisely represent the non-linear material responses of human-brain tissues, found that the peak brain-tissue strains were one order of magnitude larger than those reported by Rodríguez-Millán et al. for similar BOPs.

While several human-head FE models (Sharma, 2011; Singh et al., 2014; Garimella et al., 2018) excluded the brain-surface convolutions (i.e., the ridges and grooves on the human-brain cerebral cortex), Yu et al. (2020), who represented the gyri and the sulci to account for brain-geometry effects, found that the gyri influenced the blast-induced brain-tissue strain rates. Similarly, while the human brain is comprised of over 643,738 m of vasculature, including veins, arteries, venules, and arterioles (Begley and Brightman, 2003), with a few exceptions (Hua et al., 2015; Cotton et al., 2016; Zhao and Ji, 2020; Subramaniam et al., 2021), most FE models do not represent the cerebral vessels (Taylor and Ford, 2009; Nyein et al., 2010; Sharma, 2011; Panzer et al., 2012; Singh et al., 2014; Tan et al., 2014; Wang et al., 2014; Salimi Jazi et al., 2016; Tan et al., 2017; Garimella et al., 2018; Yu et al., 2020). Using a 3-D surrogate FE model that approximated the human head as a sphere and the blood-vessel network as tessellations, Hua et al. (2015) showed that the cerebral vasculature redistributed blast-induced brain-tissue strains by as much as 612%. However, approximations of the head and vessel geometries possibly limited the accuracy of their model-predicted responses. Moreover, while Cotton et al. (2016) reported that inclusion of anatomically accurate cerebral veins in a 3-D human-head FE model influence blast-induced brain-tissue stresses and strains, the truncated network of the represented vessels potentially limited the accuracy of their model predictions.

Recently, using a high-fidelity rat-head FE model, we showed that the inclusion of a detailed network of cerebral vessels stiffens the brain tissues, decreasing the brain-tissue strains by as much as 33% for blast-loading conditions (Unnikrishnan et al., 2019). More recently, using a high-fidelity human-head FE model, we showed that inclusion of a detailed network of cerebral veins and arteries decreased brain-tissue strains in the human brain by as much as 28% for blunt impacts (Subramaniam et al., 2021). Here, we hypothesize that inclusion of such a comprehensive network of cerebral veins and arteries, not represented in any of the human-head blast models discussed above, can further enhance model-predicted blast-induced biomechanical responses of human brain tissues and help identify correlates of blast-induced brain injury. To this end, we coupled our 3-D high-fidelity FE model of the human head, previously validated for blunt impact, with a 3-D shock-tube FE model. Using this coupled model, we simulated a human head facing an incoming blast wave and validated this enhanced human-head FE model, which includes the detailed network of cerebral veins and arteries, the gyri and the sulci, and hyper-viscoelastic brain-tissue properties, by comparing our model predictions with measurements obtained from cadaver-head experiments in a shock tube. Then, we quantified the influence of the cerebral vasculature by comparing the biomechanical responses of our detailed-vasculature model with those of a reduced-vasculature model and a no-vasculature model.

## Materials and Methods

### Finite-Element Model of the Human Head

We previously developed and validated a 3-D high-fidelity FE model of a 50th percentile United States male head to simulate blunt impact (Subramaniam et al., 2021). Here, we extended that model to simulate blast loading. Briefly, the high-fidelity FE model includes the skin, adipose tissue, eyes, sinuses, cervical spine, skull, brain, meninges, cerebral arteries, and cerebral veins (total vasculature length of 15 m). The cerebral arteries (minimum diameter of 0.24 mm) comprise the anterior cerebral arteries, middle cerebral arteries, anterior communicating artery, lenticulostriate arteries, superior and inferior cerebellar arteries, basilar artery, vertebral artery, and the detailed network of posterior communicating arteries. The cerebral veins (minimum diameter of 0.52 mm) comprise the superior and inferior sagittal sinuses, sigmoid sinus, transverse sinus, straight sinus, occipital sinus, internal vein, posterior fossa veins, deep middle cerebral veins, great cerebral vein, cerebellar veins, and the detailed network of cerebral veins.

**FIGURE 1:**
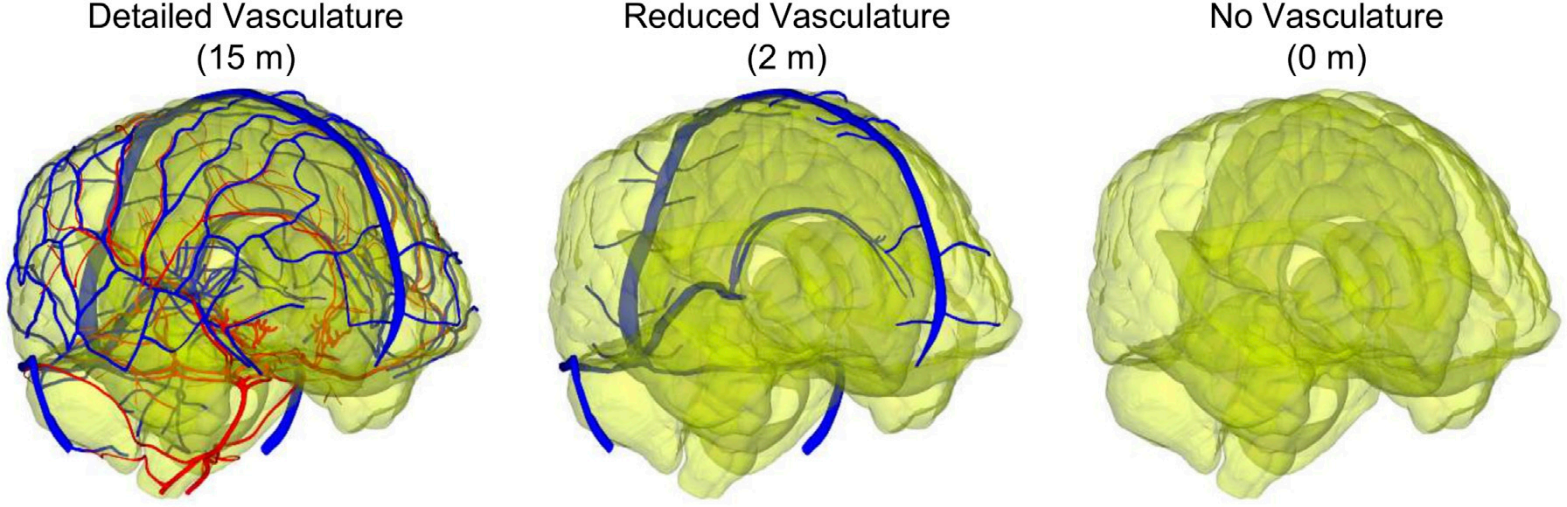
Comparison between the detailed-vasculature (15 m), reduced-vasculature (2 m), and no-vasculature (0 m) models. (Note: the arteries and veins are displayed in red and blue, respectively, whereas the brain is displayed with a transparent color.).

For the human-head FE model, we used the same mesh described in our recent study (Subramaniam et al., 2021). Briefly, we used OpenFlipper 3.1 (Möbius and Kobbelt, 2012) to mesh the individual components by creating triangular surface meshes without losing important anatomical features. Next, using Hypermesh 2017.1 (Altair Engineering, Troy, MI), we generated modified quadratic tetrahedral (C3D10M) volume meshes (total number: 4,289,775) of the skin, adipose tissue, cervical spine, skull, brain, meninges, eyes, and sinuses with an average element size of 2.3 mm, determined previously using a mesh-convergence analysis, and merged the volume meshes to prevent relative motion between different anatomical components. We then converted the vasculature surface mesh to reduced-integration (S3R) shell elements having an average element size of 0.27 mm (total number: 825,898) and assigned shell thicknesses of 0.12 and 0.10 mm to the veins and arteries, respectively. We used an embedded-element method to enforce a no-slip condition between the superficial vasculature and the subarachnoid space, and between the internal vasculature and the brain (Subramaniam et al., 2021). In addition, due to the short duration of the blast loading in this study, we used a free-neck boundary condition (Salimi Jazi et al., 2016). To quantify the influence of including the detailed network of cerebral veins and arteries, we also developed a reduced-vasculature model that consisted of the transverse sinus, straight sinus, occipital sinus, sagittal sinus, sigmoid sinus, great cerebral vein, and a truncated network of cerebral veins (total vasculature length of 2 m) and a no-vasculature model (Figure 1), similar to our previous study (Subramaniam et al., 2021).

We used the same material properties for the brain-tissues, the cerebral vessels, the skin, the eyes, the meninges, and the frontal sinus as those described in our recent study (Subramaniam et al., 2021). Briefly, for the material properties of the brain, the cerebral vasculature, and the skin tissue, we used values from previous studies that estimated the material parameters from mechanical tests performed on post-mortem human brain-tissue samples (Estes and McElhaney, 1970), freshly excised human cortical veins and arteries (Monson et al., 2003), and post-mortem human skin-tissue samples (Ottenio et al., 2015). We represented the brain tissue as a nearly incompressible, hyper-viscoelastic material using a Mooney-Rivlin model with a two-term Prony series (Mendis et al., 1995), whereas we modeled the cerebral vessels and the skin tissue as a nearly incompressible, hyperelastic material using a one-term Ogden model (Unnikrishnan et al., 2021). For the material properties of the eyes, we used values from previous studies that estimated the material parameters from mechanical tests performed on fresh human corneas (Elsheikh et al., 2007; Kok et al., 2014). We modeled the eyes and meninges as neo-Hookean solids (Subramaniam et al., 2021) and represented the frontal sinus using the ideal gas equation of state for air at atmospheric pressure (Cotton et al., 2016). Finally, we represented the skull as a compressible, linear-elastic material obtained from mechanical tests performed on post-mortem samples of human supraorbital bone (Dechow et al., 1993) and assumed that the material properties of the cervical spine were the same as those of the skull. It is important to note that the skull elastic modulus used in this study was only 8% lower than that used by Salimi Jazi et al. (2016) in their blast-simulation study. Table 1 summarizes the material properties used for the different anatomical components of the human-head FE model.

**TABLE 1:** Summary of the material properties used for the individual anatomical components included in the high-fidelity, detailed-vasculature human-head model.

Component	Density (kg/m^3^)	Elastic constants		Hyperelastic constants			Prony coefficients			
		Elastic modulus (GPa)	Poisson’s ratio	Bulk modulus (GPa)	Shear modulus (kPa)	α	g_1_	g_2_	τ_1_ (s)	τ_2_ (s)
Spine	1,412	13.76	0.29							
Skull	1,412	13.76	0.29							
Arteries	1,040			2.11	898.00	9.49				
Skin	1,040			0.04	23,900.00	16.55				
Brain	1,040			2.19	2.62		0.63	0.36	0.008	0.15
Veins	1,040			2.11	266.00	7.46				
Eyes	1,040			2.19	8.00					
Meninges	1,040			2.19	1.97					

### Finite-Element Model of the Shock Tube

**FIGURE 2:**
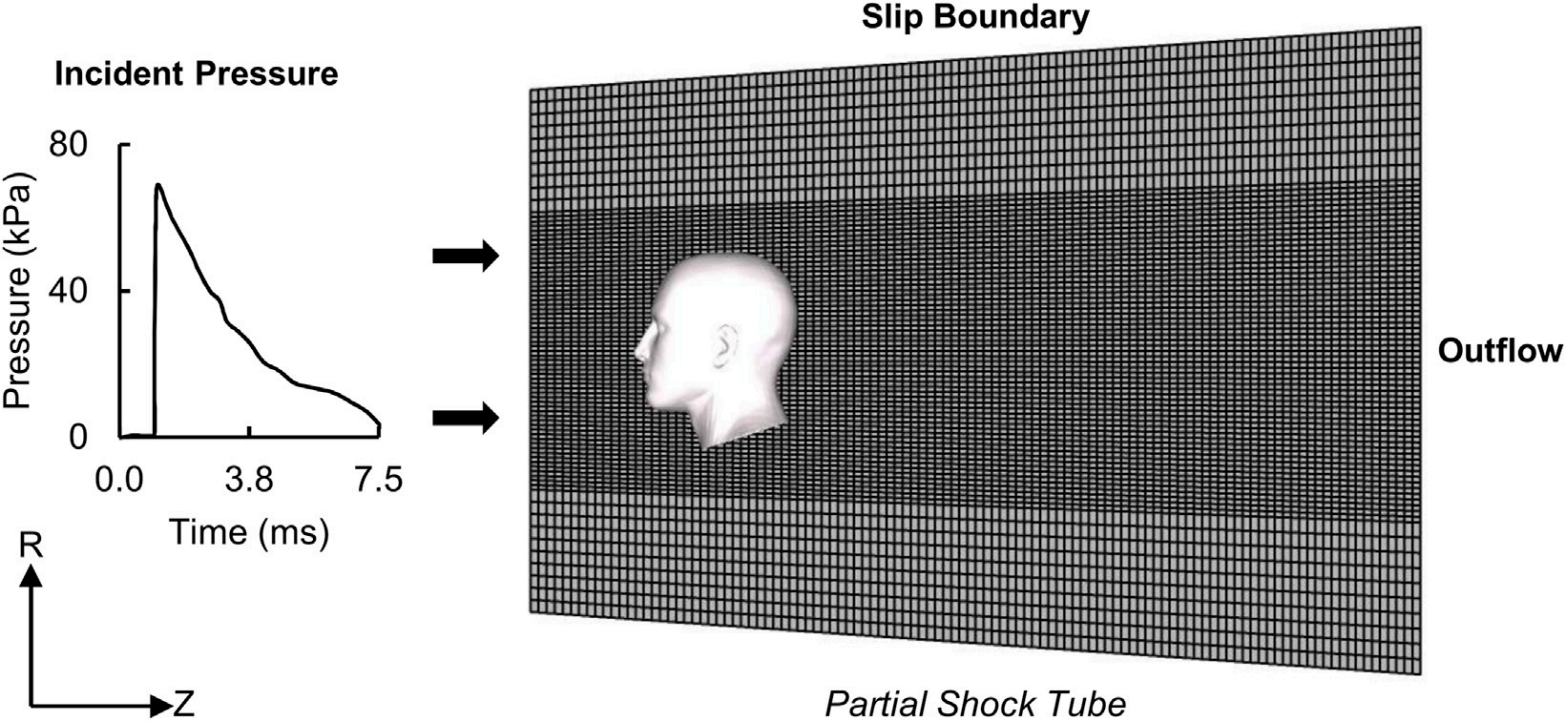
Representation of the human-head finite-element (FE) model, partial shock tube FE mesh, and boundary conditions for the blast-loading simulations. (R: radial direction; Z: axial direction.).

To simulate blast exposures in a laboratory shock tube, we used the Gmsh 4.0.6 software (Geuzaine and Remacle, 2009) to develop a 3-D FE model of a partial, 1.20-m-long diverging shock tube that had a circular cross-section with diameters of 0.71 m at the inlet and 0.87 m at the outlet. We modeled the air as an ideal gas (specific gas constant of 287 J/kg-K and density of 1.22 kg/m^3^) at a temperature of 303 K and meshed the air using 660,000 hexahedral Eulerian elements (EC3D8R). Specifically, we used a structured mesh and a biasing technique to generate finer elements for the air near the head and coarser elements elsewhere (Figure 2), and performed a mesh-convergence analysis to determine the average size for the finer elements near the head. We modeled the human head, facing the incoming blast wave, at a distance of 0.14 m from the inlet surface of the partial shock tube and coupled the shock-tube and human-head FE models using the coupled Eulerian-Lagrangian technique in ABAQUS v2018 (Dassault Systèmes Simulia Corp., Johnston, RI). Furthermore, we used a penalty contact algorithm with hard-contact normal behavior and frictionless tangential-sliding behavior to couple the Eulerian shock-tube elements with the Lagrangian human-head elements.

To simulate blast loading, we applied the measured incident pressure at the inlet surface of the partial shock tube (Figure 2) (Ganpule et al., 2013; Unnikrishnan et al., 2021). Next, to model a non-reflecting boundary, we defined an Eulerian outflow condition at the exit of the partial shock tube (Tan et al., 2014). Finally, to prevent airflow in the radial direction and constrain the blast wave to travel within the shock tube in the axial direction, we defined a slip boundary condition at the shock-tube wall (Yu and Ghajari, 2019). We performed all simulations using ABAQUS/Explicit on a SGI 8600 system termed Koehr at the United States Navy Department of Defense Supercomputing Resource Center and on a SGI 8600 system termed Mustang at the United States Air Force Research Laboratory Supercomputing Resource Center. Using 48 CPU cores and a stable time step of 21 ns determined by the double-precision ABAQUS solver, we completed 7.5-ms simulations in 60 h. Overall, we performed five simulations for three BOPs (detailed-vasculature model: 1 per BOP, reduced- and no-vasculature models: 1 each for the high-intensity BOP).

### Experimental Study for Model Validation

To evaluate our high-fidelity FE model for blast loading, we used the measured biomechanical responses reported by Leonardi (2012) and Bir (2011), who performed experiments in a shock tube with the characteristics discussed above on cadaver heads for three BOPs: 1) low-intensity BOP (68 kPa), 2) medium-intensity BOP (83 kPa), and 3) high-intensity BOP (104 kPa). They perfused one male and three female heads, obtained from fresh, unembalmed cadavers (mean age ± one standard deviation: 75.0 ± 16.5 years), with artificial cerebrospinal fluid at a constant pressure of 10.2 kPa. Then, they placed the head upside down in a soft net, suspended it near the center of the expansion section at approximately 1.25 m from the open end of the diverging shock tube, and delivered the BOP to the head using a shock-wave generator (Bir, 2011; Leonardi, 2012).

For each cadaver head and each BOP, the authors performed blast-exposure tests in the frontal, occipital, and two lateral orientations, where for each test they measured the intracranial pressure in the right frontal lobe, the right lateral ventricle, the right parietal lobe, and the right occipital lobe. However, we only used the test results for the frontal orientation because this was the only case for which they performed one repeated measurement per cadaver for each BOP. In addition, we only used measurements at the frontal lobe and the ventricle because of the large observed differences in the pressure-time profile between cadavers in the other two measurement locations. Finally, to measure the blast-induced skull strains, the authors placed rosette strain gages on the left frontal bone, the left occipital bone, the left zygomatic bone, the left sphenoid bone, and the left parietal bone. Nevertheless, we only compared the results for measurements at the left frontal bone because for the other locations the authors did not report the maximum principal strain (MPS) for each cadaver due to sensor failure and data-acquisition loss.

### Analysis and Comparison

To post-process the simulation results, we used the EnSight 10.2.5a software (Computational Engineering International, Inc., Apex, NC) to summarize the intracranial pressure (ICP), the von Mises stress (VMS), the strain rate, and the MPS. We evaluated the ICP because primary blast loads are known to change the balance among the intracranial content volumes and increase the ICP (Elder and Cristian, 2009), generating volumetric tension in the brain tissue and subsequently causing axonal damage (Taylor and Ford, 2009). In addition, blast-induced shear stress can possibly cause concussion (Taylor and Ford, 2009), axonal stretching could potentially damage the brain white matter (Bain and Meaney, 2000), and high strain rates could possibly influence the pathomorphology of neuronal injury (Bar-Kochba et al., 2016). Consistent with our previous study (Subramaniam et al., 2021), we used the model-predicted values of VMS and MPS as surrogates for blast-induced shear stress and axonal stretch, respectively. Next, using the Correlation and Analysis (CORA) software (Gehre et al., 2009), we compared the simulated and experimental pressure-time profiles. For these comparisons, we used the cross-correlation analysis module to evaluate the size, progression, and phase shift of the simulated pressure-time profile with respect to the experimental measurements and the corridor analysis module to evaluate the deviation between the simulated and experimental pressure-time profiles. For each of these modules, we used the corresponding default software parameter values.

## Results

### Model Convergence

We performed mesh-convergence tests on the shock-tube FE model using four mesh configurations to determine the adequate number of mesh elements (Table 2). To this end, we systematically increased the number of elements and evaluated the maximum air pressure at the nasal bridge. We observed a difference of 8.5% in the maximum pressure between a model with 179,200 elements (T1 in Table 2) and a model with 405,000 elements (T2 in Table 2). Conversely, we observed a pressure difference of only 2.5% between the current model with 660,000 elements (T3 in Table 2) and T2. Moreover, the peak pressure predicted by the current model increased marginally (1.1%) when we increased the number of elements to 1,001,000 (T4 in Table 2), indicating that we achieved convergence with the T3 model.

**TABLE 2:** Summary of the mesh-convergence tests for the shock-tube model.

Model	Elements	Element size (mm)	Maximum air pressure (kPa)
T1	179,200	10.0	104.4
T2	405,000	8.0	113.3
T3^a^	660,000	6.0	116.1
T4	1,001,000	4.0	117.4

a Selected.

### Model Validation

Using the frontal blast-wave exposure data from Bir (2011) and Leonardi (2012), we validated our detailed-vasculature FE model for BOPs of 68, 83, and 104 kPa by comparing the model-predicted and experimentally measured ICP values at the right frontal lobe and the right lateral ventricle. It is important to note that Leonardi (2012) discarded 1, 3, and 4 frontal-lobe pressure recordings for the 68, 83, and 104 kPa BOPs, respectively, because sensor malfunction, loss of tracking, and blast-wind noise limited the reliability of these measurements. In addition, Leonardi did not report ventricle pressure recordings for the male cadaver corresponding to the 83 and 104 kPa BOPs due to sensor failure. Therefore, to evaluate our model, we used 7, 5, and 4 frontal-lobe pressure measurements and 8, 6, and 6 ventricle pressure measurements, with durations of up to 6 ms each, for the 68, 83, and 104 kPa BOPs, respectively, as reported by Leonardi (2012) (Appendix A, page 160–249).

**FIGURE 3:**
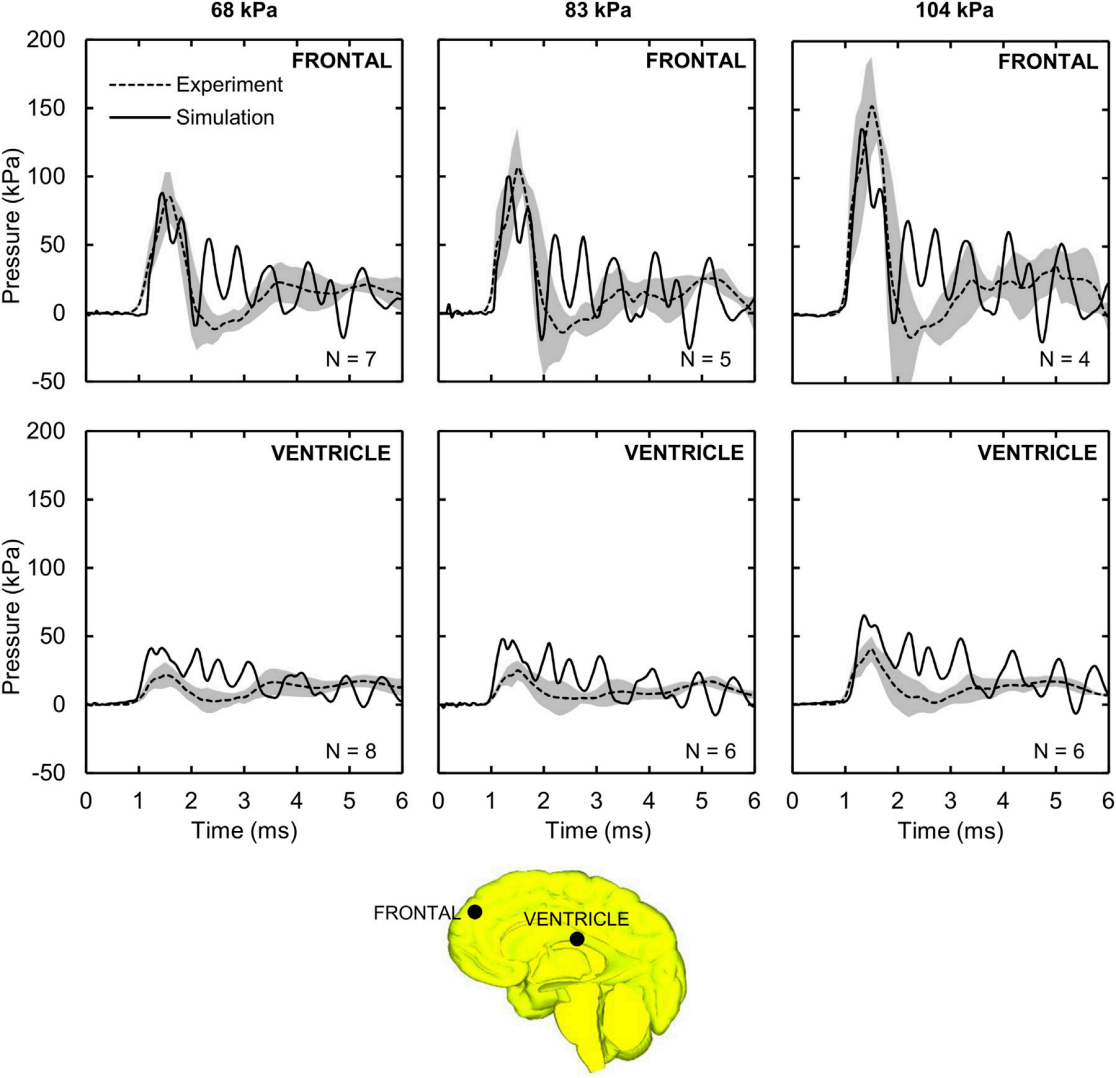
Intracranial pressure (ICP) measured in the shock-tube experiment and predicted by the detailed-vasculature model. Shown are the temporal profile of the predicted (solid lines) and experimentally observed (dashed lines) ICP values at the frontal lobe and ventricle for the 68, 83, and 104 kPa blast overpressures. Shaded regions; mean ± two standard errors of the mean. (Locations selected for comparison are indicated by black circles on the mid-sagittal brain geometry.).

Figure 3 shows the measured and predicted temporal profiles of the ICP values at the two locations, and Table 3 summarizes the CORA ratings for the three BOPs. From 0 to about 2 ms, the model-predicted ICP in the frontal lobe increased and then decreased with time, matching well with the experimental measurements and yielding peak-magnitude differences of 4, 6, and 11% and phase-shift differences of 9, 10, and 13% for the 68, 83, and 104 kPa BOPs, respectively. After this initial phase, the experimentally measured ICP decreased to a negative value and then gradually increased with time at similar rates for each of the three BOPs, while the model-predicted values oscillated with time, possibly due to reflections from the skin-skull, skull-subarachnoid space, and subarachnoid space-brain interfaces and deformation of the skull. However, when we computed the CORA ratings for up to 3 ms, we found that each of the model predictions in the frontal lobe yielded a good bio-fidelity rating (0.67–0.74), with differences in CORA ratings of less than 11% among the three pressures (Table 3). In contrast, the model predictions for up to 6 ms yielded a fair bio-fidelity rating (0.52–0.56), with differences of less than 8%.

**TABLE 3:** Correlation and analysis (CORA) rating between the simulated and experimental pressure-time profiles.

Location/Time (ms)	Blast overpressure (kPa)			Average
	68	83	104	
Frontal lobe				
3	0.74	0.69	0.67	0.70
6	0.52	0.56	0.54	0.54
Ventricle				
3	0.42	0.37	0.50	0.43
6	0.33	0.32	0.42	0.36

Bio-fidelity scale for CORA rating: 0.86–1.00, excellent; 0.65–0.86, good; 0.44–0.65, fair; 0.26–0.44, acceptable; 0.00–0.26, unacceptable.

The model-predicted ventricle pressure oscillated throughout the simulation time (Figure 3), presumably due to reflections from the skin-skull, skull-subarachnoid space, and subarachnoid space-brain interfaces and the elastic response of the meninges. In contrast, the experimentally measured pressure increased with time, reaching a peak between 21.5 and 40.6 kPa around 1.5 ms, and then slowly decreased and increased. We observed phase-shift discrepancies between the simulation and experimental results for the first pressure peak (at ∼1.5 ms) of 17, 20, and 10%, and peak-pressure differences of 91, 90, and 61% for the 68, 83, and 104 kPa BOPs, respectively. Despite these differences, when we computed the CORA ratings for up to 3 ms, we found the model predictions to be fair for the 104 kPa BOP, but only acceptable for the 68 and 83 kPa BOPs (Table 3). In contrast, the CORA ratings for up to 6 ms ranged from 0.32 to 0.42, indicating that the model predictions were acceptable for all three BOPs.

### Amplification of the Incident Pressure

**FIGURE 4:**
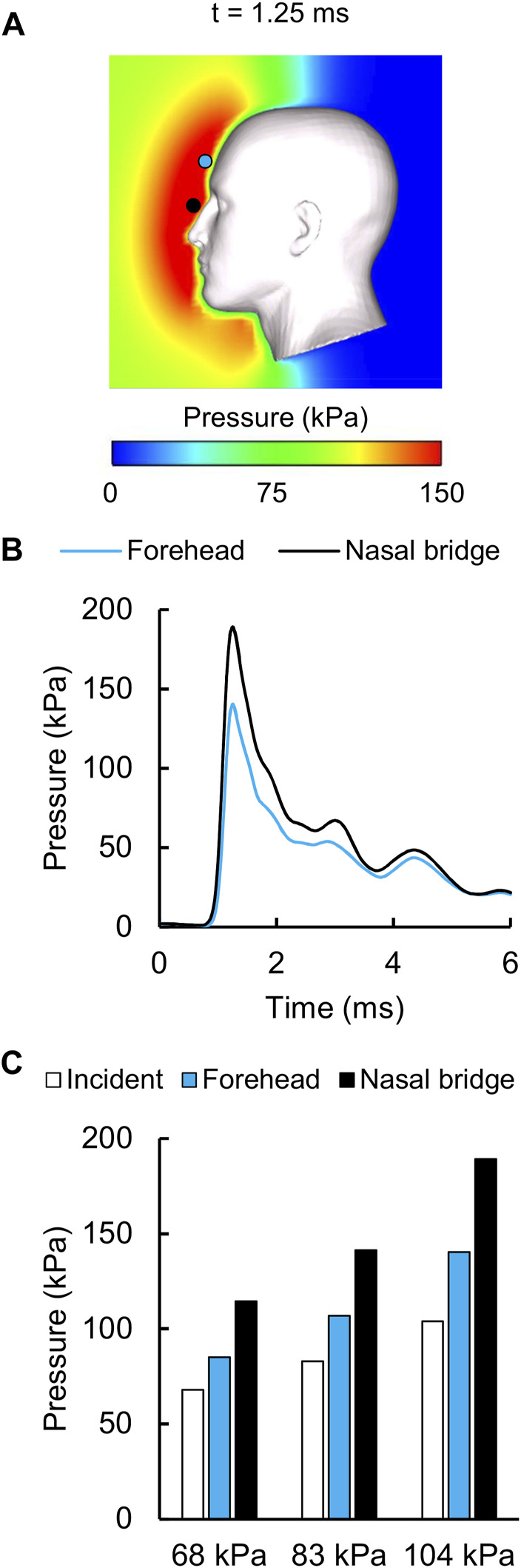
Reflected air pressure near the human head in the shock tube. **(A)** Contour map showing the model-predicted peak reflected pressure for the 104 kPa blast overpressure at t = 1.25 ms into the simulation. **(B)** Temporal profile of the predicted reflected pressure at the nasal bridge (black circle) and the forehead (blue circle). **(C)** Bar graph comparing the measured incident pressures and the predicted reflected pressures for the 68, 83, and 104 kPa blast overpressures.

For the detailed-vasculature model, we evaluated the amplification (i.e., the reflection) of the incident pressure resulting from the interaction between the blast wave and the head. Figure 4A shows the distribution of the reflected pressure for the 104 kPa BOP resulting from the blast-wave diffraction at 1.25 ms into the simulation. In addition, Figure 4B shows the temporal profile of the reflected pressure at the nasal bridge and the forehead for the 104 kPa BOP, and Figure 4C compares the measured peak incident pressures and the predicted reflected pressure values for the three BOPs. Compared to the incident pressures of 68, 83, and 104 kPa, respectively, the peak reflected pressures were 68, 70, and 82% higher at the nasal bridge and 25, 29, and 35% higher at the forehead.

### Model-Predicted Von Mises Stress, Strain Rate, and Maximum Principal Strain

**FIGURE 5:**
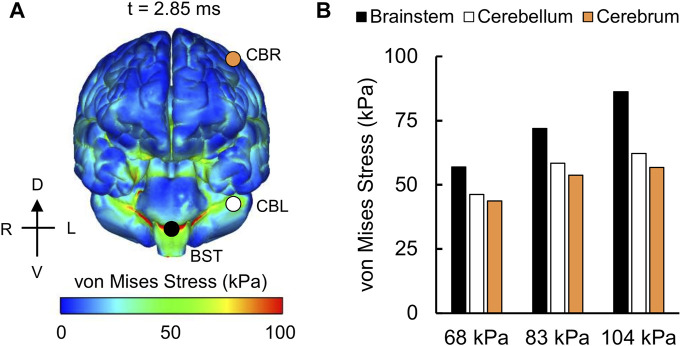
Simulation showing the variation of the von Mises stress as a function of brain location. **(A)** Contour map showing the model-predicted von Mises stress distribution throughout the outer surface of the brain for the 104 kPa blast overpressure at t = 2.85 ms into the simulation. CBR: cerebrum (orange circle); CBL: cerebellum (white circle); BST: brainstem (black circle). **(B)** Bar graph comparing the peak von Mises stresses for the 68, 83, and 104 kPa blast overpressures. D: dorsal; V: ventral; R: right; L: left.

**FIGURE 6:**
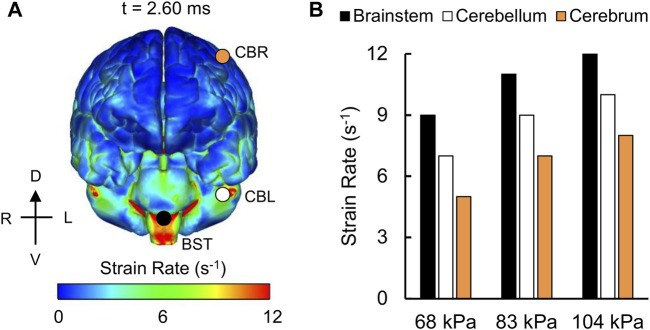
Simulation showing the variation of the strain rate as a function of brain location. **(A)** Contour map showing the model-predicted strain-rate distribution throughout the outer surface of the brain for the 104 kPa blast overpressure at t = 2.60 ms into the simulation. CBR: cerebrum (orange circle); CBL: cerebellum (white circle); BST: brainstem (black circle). **(B)** Bar graph comparing the peak strain rates for the 68, 83, and 104 kPa blast overpressures. D: dorsal; V: ventral; R: right; L: left.

**FIGURE 7:**
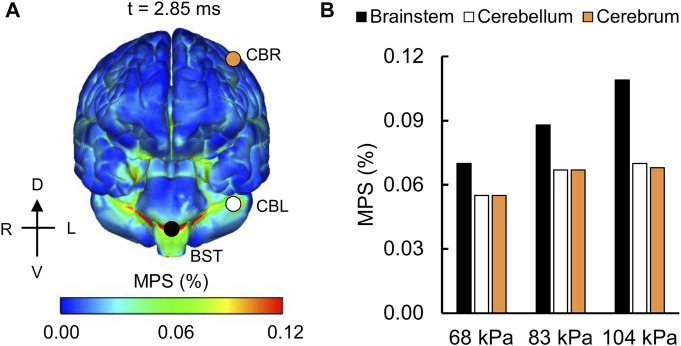
Simulation showing the variation of the maximum principal strain (MPS) as a function of brain location. **(A)** Contour map showing the model-predicted MPS distribution throughout the outer surface of the brain for the 104 kPa blast overpressure at t = 2.85 ms into the simulation. CBR: cerebrum (orange circle); CBL: cerebellum (white circle); BST: brainstem (black circle). **(B)** Bar graph comparing the peak MPS for the 68, 83, and 104 kPa blast overpressures. D: dorsal; V: ventral; R: right; L: left.

For the detailed-vasculature model, we compared the peak VMS, strain rate, and MPS at three locations: 1) cerebrum, 2) cerebellum, and 3) brainstem. We observed higher VMS values in the brainstem compared to the cerebellum and cerebrum (Figure 5A), with the peak VMS occurring at 2.85 ms in the brainstem for the 104 kPa BOP. For the three BOPs, the peak VMS values ranged from 57 to 86 kPa in the brainstem, from 46 to 62 kPa in the cerebellum, and from 44 to 57 kPa in the cerebrum (Figure 5B). For the 104 kPa BOP, we observed higher strain rates in the brainstem and cerebellum compared to the cerebrum (Figure 6A), with the peak strain rate occurring at 2.60 ms in the brainstem. The peak strain-rate values ranged from 9 to 12 s^−1^ in the brainstem, from 7 to 10 s^−1^ in the cerebellum, and from 5 to 8 s^−1^ in the cerebrum (Figure 6B). In terms of MPS, we observed higher values in the brainstem compared to the cerebellum and cerebrum (Figure 7A), with the peak MPS occurring at 2.85 ms in the brainstem for the 104 kPa BOP. For the brainstem, the peak MPS values ranged from 0.070 to 0.110% across the three BOPs, whereas for the cerebellum and cerebrum, they ranged from 0.055 to 0.070% (Figure 7B).

### Influence of the Vasculature on Model Predictions

**FIGURE 8:**
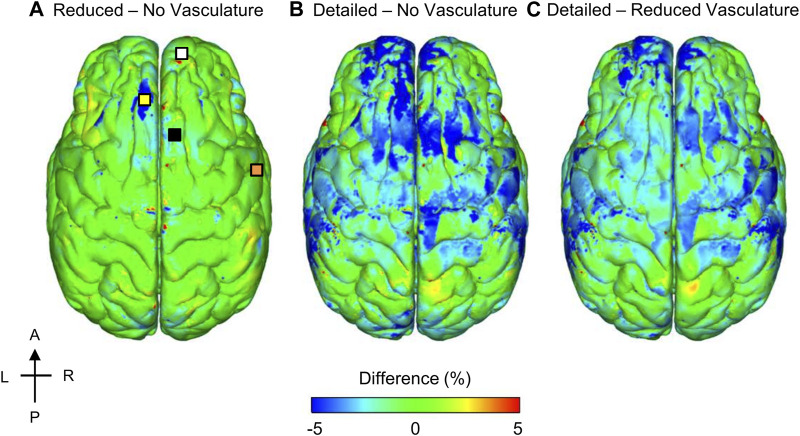
Differences in the peak maximum principal strain (MPS) between models for the 104 kPa blast overpressure. Shown are the difference maps comparing the peak MPS between the reduced- and no-vasculature models **(A)**, between the detailed- and no-vasculature models **(B)**, and between the detailed- and reduced-vasculature models **(C)**. For each pair of comparisons, we computed differences in model predictions by first determining the peak MPS for each model at each tetrahedral element of the human brain over the blast-exposure simulation time, and then subtracting them. Locations selected for comparison include: frontal lobe (white square), longitudinal fissure (yellow square), parieto-frontal brain (black square), and temporal lobe (orange square) on the reduced – no vasculature difference map. A: anterior; P: posterior; R: right; L: left.

To quantify the influence of the vasculature, we compared the model-predicted ICP values corresponding to the detailed-, reduced-, and no-vasculature models for the 104 kPa BOP and observed that, as expected, there were no differences in the magnitude or time course of the simulated ICP for the three models. This is because while the inclusion of vasculature increases the brain stiffness, it does not change its compressibility. Next, to investigate the importance of the cerebral vasculature in the estimation of the MPS, we generated 3-D difference maps by comparing the reduced-vasculature model with the no-vasculature model, the detailed-vasculature model with the no-vasculature model, and the detailed-vasculature model with the reduced-vasculature model (Figure 8). Specifically, for each pair of models, we first determined the peak MPS for each model at each tetrahedral element of the brain over the blast-exposure simulation time, and then subtracted them, similar to our previous study (Rubio et al., 2020). The peak MPS values in the frontal lobe (white square marker) were comparable for the reduced- and no-vasculature models, but 6% lower for the detailed-vasculature model. In contrast, the peak MPS values in the longitudinal fissure (yellow square marker) were comparable for the detailed- and reduced-vasculature models, but 7 and 5% lower, respectively, than that for the no-vasculature model. Compared to the detailed-vasculature model, the peak MPS values in the parieto-frontal brain (black square marker) were 3 and 5% higher for the reduced- and no-vasculature models, respectively. The peak MPS values in the temporal lobe (orange square marker) were comparable for the reduced- and no-vasculature models but 7% lower for the detailed-vasculature model.

**FIGURE 9:**
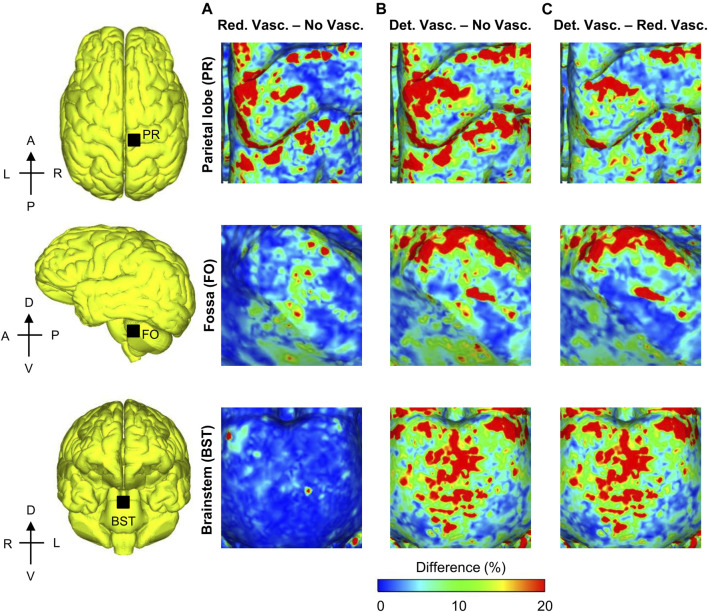
Differences in the peak strain gradient between models for the 104 kPa blast overpressure for three regions in the brain. Shown are the difference maps comparing the peak strain gradient between the reduced- and no-vasculature models **(A)**, between the detailed- and no-vasculature models **(B)**, and between the detailed- and reduced-vasculature models **(C)**. For each pair of comparisons, we computed differences in model predictions by first determining the peak strain gradient for each model at each tetrahedral element of the human brain over the blast-exposure simulation time, and then subtracting them. Det. Vasc.: detailed-vasculature model; Red. Vasc.: reduced-vasculature model; No Vasc.: no-vasculature model; A: anterior; P: posterior; R: right; L: left; D: dorsal; V: ventral.

To quantify the re-distribution of the strain as a function of the vasculature represented in the models, we evaluated the spatial gradient of the peak MPS values for the detailed-vasculature model and generated 3-D difference maps to compare the model-predicted strain gradient with those obtained from the reduced- and no-vasculature models (Figure 9). Specifically, for each pair of models, we first determined the peak strain gradient for each model at each tetrahedral element of the brain over the blast-exposure simulation time, and then subtracted them. The peak strain gradients in the parietal lobe sulcus and the longitudinal fissure were comparable for the detailed- and reduced-vasculature models, with a 30% higher peak strain gradient than that of the no-vasculature model. In contrast, the peak strain gradients in the fossa and the brainstem were comparable for the reduced- and no-vasculature models but were 30% higher for the detailed-vasculature model.

### Model-Predicted Maximum Principal Strain at the Skull

**FIGURE 10:**
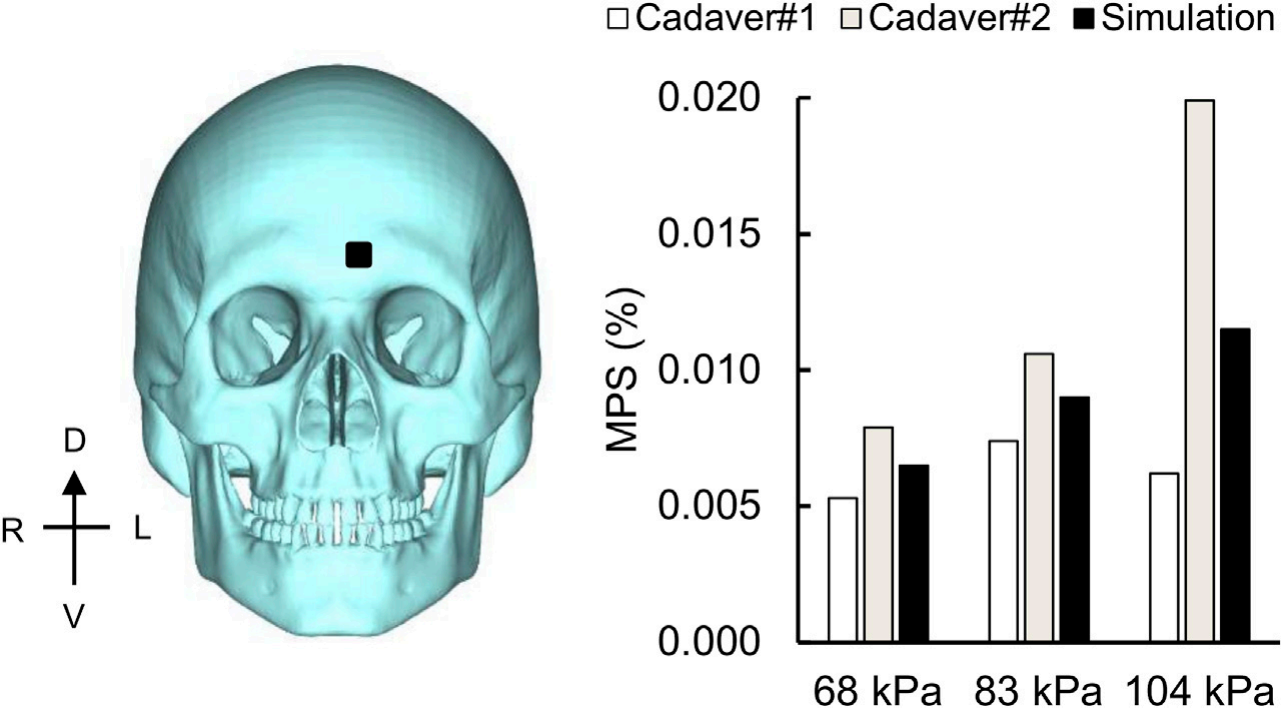
Simulation showing the maximum principal strain (MPS) on the skull. Bar graph comparing the simulated and experimentally measured peak MPS values on the frontal bone (black square) for the 68, 83, and 104 kPa blast overpressures. D: dorsal; V: ventral; R: right; L: left.

Using the frontal blast-wave exposure data from Bir (2011) and Leonardi (2012), we compared the experimentally measured peak MPS values and the model predictions on the left frontal bone (Figure 10), similar to a previous study (Sharma, 2011). For the 68, 83, and 104 kPa BOPs, while the experimentally measured peak MPS values for two cadavers ranged from 0.005 to 0.020%, the simulated values varied between 0.007 and 0.012%. However, when we compared the simulated values with the mean of the two measurements, we observed differences of less than 12% for all three BOPs.

## Discussion

Using a 3-D high-fidelity FE model of a human head, we evaluated the influence of the cerebral vasculature on the biomechanical responses of brain tissues during exposure to a blast wave in a shock tube. To this end, we coupled our previously developed human-head FE model, which we validated for blunt impact (Subramaniam et al., 2021), with a 3-D FE model of a diverging shock tube. Then, we used the coupled model to determine the biomechanical responses of brain tissue to frontal blast-wave loading for BOPs equivalent to 68, 83, and 104 kPa. We validated the FE model for each of the three BOPs by comparing the model-predicted ICP values at the right frontal lobe and the right lateral ventricle of the human brain with those obtained from cadaver heads exposed to the same BOPs in a shock tube. In our model, we included a detailed network of cerebral veins and arteries to more comprehensively model the brain-tissue stiffness and the resulting blast-induced biomechanical responses. To evaluate this enhancement for blast loading, we compared and contrasted the model-predicted ICP and MPS values with those obtained from a reduced-vasculature model and a model with no vasculature for the same BOP.

### Comparison of Model Features

The three key attributes of our 3-D high-fidelity human-head FE model are 1) the detailed network of cerebral veins and arteries (Figure 1), 2) the representation of the brain-tissue gyri and sulci, and 3) the hyper-viscoelastic material properties to model blast-induced brain-tissue deformations (Table 1). In contrast, Sharma (2011), Singh et al. (2014), and Panzer et al. (2012), who developed coupled FE models similar to our study, and Garimella et al. (2018), who applied a blast load to a human-head FE model using the Conventional Weapons Program (Hyde, 1991), did not model the cerebral vasculature or the brain-tissue gyri and sulci. Moreover, Sharma (2011), Singh et al. (2014), and Panzer et al. (2012) employed linear viscoelastic brain-tissue properties, whereas Garimella et al. (2018) used hyper-viscoelastic material properties to model blast-induced brain-tissue deformations. In turn, while the coupled FE models developed by Ganpule et al. (2013), Wang et al. (2014), Taylor and Ford (2009), and Nyein et al. (2010) included the gyri and sulci, they did not model the cerebral vasculature. In addition to these methodological differences, there are also differences in the geometry of the modeled human brain. For example, while we used the geometry of a 50th percentile United States male head, Taylor and Ford developed their model using a female-head geometry. We infer that the variations in the blast-induced biomechanical responses of brain tissues between our human-head FE model and previously developed models are due to the above-mentioned differences in modeling representation.

### Model Validation and Pressure Amplification

For the frontal lobe, we observed good agreement between the model-predicted and experimental ICP values, with peak-pressure discrepancies of less than 12% and temporal phase-shift differences of less than 0.25 ms for the three BOPs simulated in our study (Figure 3). Previous model validations have reported similar or larger discrepancies between simulations and experiments. For instance, Sharma (2011) and Singh et al. (2014) reported peak-pressure discrepancies in the frontal lobe that varied from 5 to 30% and temporal phase-shift differences of less than 0.50 ms for BOPs ranging from 71 to 104 kPa. In contrast, Garimella et al. (2018) observed peak-pressure discrepancies that varied between 25 and 50% for BOPs ranging from 71 to 104 kPa. The larger peak-pressure discrepancy between our study and Garimella et al. could be due to the differences in the algorithm implemented to apply the blast load to the head. Similar to Sharma (2011) and Garimella et al. (2018), we observed oscillations in the simulated pressure-time profiles compared to the experimental values. These oscillations in the frontal lobe could be possibly attributed to the blast wave continuously deforming the skull as it propagates through the head (Garimella et al., 2018; Moss et al., 2009) as well as reflections from the skin-skull, skull-subarachnoid space, and subarachnoid space-brain interfaces (Ganpule et al., 2013). To investigate the potential reasons for our model-predicted pressure oscillations, we performed numerous simulations using different material properties for the skull (elastic modulus), skin (bulk modulus), and cerebrospinal fluid (shear modulus) from the nominal values in Table 1. However, when we changed each of these properties one at a time while considering a range of values around the nominal value, we observed no changes in the amplitude or frequency of the oscillations in the model predictions. Hence, we believe that our choice of material properties was not the cause of the observed pressure oscillations.

Despite the differences between the experimental and simulated pressure-time profiles, the CORA rating scored our model predictions as fair in the frontal lobe for the 104 kPa BOP (Table 3). This compares favorably with the model developed by Sharma (2011) (acceptable score) and the model by Garimella et al. (2018) (unacceptable score) for the same BOP, suggesting that our high-fidelity model offered better ICP predictions in the frontal lobe when compared to these models. Furthermore, we did not observe any differences in the ICP values for the detailed-, reduced-, and no-vasculature models, similar to our previous studies (Unnikrishnan et al., 2019; Subramaniam et al., 2021). This is because the inclusion of the vasculature increases the brain shear modulus but does not change its bulk modulus (Unnikrishnan et al., 2019).

The peak-pressure discrepancy in the ventricle (61%) for the 104 kPa BOP was comparable to the value reported by Sharma (2011), who observed a peak-pressure discrepancy equivalent to 50%. We observed oscillations in the predicted pressure-time profiles, similar to those in Sharma’s model, possibly due to reflections from the skin-skull, skull-subarachnoid space, and subarachnoid space-brain interfaces and the elastic response of the meninges. In contrast, we did not observe such oscillations in the experimental measurements. We hypothesize that the artificial cerebrospinal fluid used to perfuse the cadavers could have minimized the blast-induced oscillations in the ventricle. Despite these differences between the simulated and experimental pressure-time profiles, we found that the CORA rating was acceptable in the ventricle for the 104 kPa BOP, consistent with Sharma’s model. We observed an amplification of the incident pressure due to blast-wave diffraction (Figure 4), consistent with Ganpule et al. (2013) and Sharma (2011). In particular, for the 104 kPa BOP, the peak reflected pressure in our model (189 kPa) matched well with the predicted value reported by Sharma (180 kPa). In addition, we observed that the peak reflected pressure was at the nasal bridge, similar to the location reported by Ganpule et al. However, unlike their model, which predicted that the reflected pressure was 140% higher than the 230 kPa incident pressure, we found that the difference between the reflected and incident pressures did not exceed 82% for the 104 kPa BOP. This discrepancy between the two models could be due to differences in the incident pressure intensities (104 vs. 230 kPa, the only reported value), boundary conditions, head geometry, and perhaps the shock-tube geometry. Interestingly, after normalizing the experimental incident pressure to 230 kPa, we found that their model prediction (Ganpule et al., 2013) was 25% lower than their experimentally measured reflected pressure at the forehead for 200 kPa (Ganpule, 2013; Chandra and Sundaramurthy, 2015). Similarly, when we compared our model-predicted peak reflected pressure for the 68 kPa BOP case and the measurements obtained from cadaver experiments at 70 kPa (Ganpule, 2013; Chandra and Sundaramurthy, 2015), we found that our model prediction was 45% lower than the experimental value.

### Von Mises Stress, Strain Rate, and Maximum Principal Strain

Overall, the peak brain-tissue VMS, strain rate, and MPS values predicted by our detailed-vasculature model ranged from 44 to 86 kPa, 5 to 12 s^−1^, and 0.055–0.110%, respectively, for BOPs of 68, 83, and 104 kPa (Figures 5–7). These results also showed that the inclusion of additional vasculature decreased the brain-tissue strain by 7% (Figure 8) and redistributed it by as much as 30% in the proximity of the vessels (Figure 9). For the detailed-vasculature model, we observed higher VMS in the brainstem compared to the cerebrum (Figure 5), consistent with the work of Wang et al. (2014). We observed peak VMS values ranging from 44 to 57 kPa in the cerebrum for the three BOPs, which was within the range of values reported previously (Taylor and Ford, 2009; Nyein et al., 2010). For example, while Taylor and Ford (2009) observed a peak VMS equivalent to 25 kPa, Nyein et al. (2010) reported a peak VMS value of 600 kPa in the cerebrum for frontal blast loading. For the detailed-vasculature model, we observed peak strain rates ranging from 5 to 8 s^−1^ in the cerebrum for the three BOPs simulated in our study (Figure 6), whereas Panzer et al. (2012) reported peak strain rate values that varied between 12 and 22 s^−1^ for BOPs ranging from 50 to 100 kPa. We believe that the differences in the predicted strain rate values between the two models most likely resulted from different selections of brain-tissue properties. For the detailed-vasculature model, we observed higher MPS in the brainstem compared to the cerebrum and cerebellum (Figure 7), consistent with the work of Sharma (2011). We observed peak MPS ranging from 0.06 to 0.11% for the three BOPs simulated in our study, whereas Sharma reported peak MPS values that varied between 0.50 and 5.00% for BOPs ranging from 71 to 104 kPa. We believe that the differences in the predicted MPS values between the two models could be due to the selection of the viscoelastic brain-tissue properties.

While the reduction in peak MPS due to inclusion of the cerebral vasculature (Figure 8) was consistent with our previous study describing a high-fidelity rat-head FE model (Unnikrishnan et al., 2019), the amount by which the strain decreased was different for the human-head model when compared to the rat-head model. Incorporating the detailed human cerebral vessels decreased the human cerebrum MPS values by 7%, whereas inclusion of the rat vasculature reduced the rat cerebrum strains by 17%, possibly due to the difference in vasculature thicknesses. The localized strain reduction and redistribution of MPS depend on the amount of vasculature included in the model. For example, compared to the no-vasculature model, inclusion of the bridging veins reduced the peak MPS in the reduced-vasculature model by 5%, while inclusion of the cerebral arteries in the detailed-vasculature model reduced the MPS in the longitudinal fissure by an additional 2% (Figure 8). Furthermore, we observed strain-gradient differences of up to 30% in the fossa when we compared the detailed-vasculature model with the reduced-vasculature model (Figure 9). We attributed these large strain-gradient differences to the amount of additional cerebellar vasculature represented in the detailed-vasculature model, which included both the cerebellar veins and arteries. In contrast, as the amount of vasculature in the parietal lobe sulcus was comparable in both models, we observed smaller strain-gradient differences when we compared values at this location.

We believe that the gradient of the MPS can potentially serve as a biomechanical index that correlates with observed brain-tissue changes, similar to the study by Zhang et al. (2013), who used strain and its derivatives to assess the likelihood of bTBI from open-field blast loading. Interestingly, the depths of the parietal lobe sulci, where our detailed- and reduced-vasculature models predicted higher strain gradients when compared to the no-vasculature model, showed phosphorylated tau pathology in post-mortem analyses of human subjects with bTBI (McKee and Robinson, 2014). Overall, the predicted peak MPS values on the frontal bone varied between 0.007 and 0.012% for BOPs of 68, 83, and 104 kPa and were within the range of the measurements for the two cadavers (Figure 10). In contrast, Sharma (2011) reported peak MPS values ranging from 0.009 to 0.018% for BOPs ranging from 71 to 104 kPa. We believe that the differences in the predicted MPS values between the two models most likely resulted from different selections of skull material properties.

### Study Limitations

Our study has several limitations. First, we evaluated the influence of the vasculature for frontal blast loading only, necessarily excluding occipital and lateral blast-loading scenarios. While the head orientation influences brain VMS and MPS values (Wang et al., 2014; Unnikrishnan et al., 2021), we believe that the redistribution of MPS observed in our study will remain valid for occipital and lateral blast-wave exposure. Second, we did not model cerebral veins and arteries with diameters less than 0.52 and 0.24 mm, respectively. While we believe that the inclusion of smaller vessels would change the brain shear modulus, and subsequently modify the values of VMS and MPS, we would not expect to observe changes in the model-predicted ICP values because the inclusion of the vasculature does not change the brain bulk modulus. It is also important to note that different individuals may have different geometry of the circle of Willis, however, we do not believe that such variations will drastically change our conclusions. Third, to model the brain-tissue stiffening effect arising from the inclusion of the cerebral vasculature, we used the embedded-element method to couple the brain and the subarachnoid space with the superficial and the internal vasculature, respectively, and did not specify the blood pressure in our FE model, similar to our previous study (Subramaniam et al., 2021). Moreover, while the peak frontal lobe pressures for the 68, 83, and 104 kPa BOPs were higher than the systolic arterial blood pressure by 633, 730, and 1,028% (Dunn, 2002), respectively, we do not believe that the inclusion of blood pressure would significantly change the model-predicted ICP values. Furthermore, while it is known that the embedded-element method increases the mass in the FE model due to volume redundancy (Garimella et al., 2019), the additional mass resulting from the vasculature was only 0.06% of the total mass of the human head, implying that the potential effect of the added mass was insignificant. Fourth, in the current form, we cannot use our model to accurately predict the ventricle ICP, as the material properties of the artificial cerebrospinal fluid used to perfuse the cadavers are not available. While the meninges could be possibly modeled as a viscous fluid with the material properties of water (Singh et al., 2014), we do not expect such properties to significantly modify the simulated pressure-time profiles because other studies that approximated the meninges using the Mie-Gruneisen equation of state for water observed oscillations in the model-predicted ICP for blast-loading conditions (Garimella et al., 2018; Tong et al., 2019). Finally, we assumed homogeneous properties for the brain tissue and necessarily excluded rate-dependent material properties specific to brain white matter (Tse et al., 2017; Ramzanpour et al., 2020), which could possibly influence VMS and MPS values. Nonetheless, as the redistribution of MPS resulting from the inclusion of vasculature is consistent for homogeneous and heterogeneous brain-tissue properties (Zhao and Ji, 2020; Subramaniam et al., 2021), we expect our overall findings to remain valid.

## Conclusion

To conclude, we coupled a high-fidelity 3-D FE model of the human head, previously validated for blunt impact, with a 3-D shock-tube FE model and characterized the biomechanical responses of the brain to primary blast-wave exposure. In the FE model, we used the hyper-viscoelastic properties of human brain tissues and represented the detailed network of cerebral veins and arteries, the gyri, and the sulci. As expected, the cerebral vasculature did not influence the pressure response of the brain, but influenced the shear response, redistributing the brain-tissue strains by as much as 30% in the proximity of the vessels, the gyri, and the sulci. These observations suggest that the more detailed network of cerebral vasculature and the brain-surface convolutions included in our high-fidelity FE model considerably influence certain biomechanical responses of the brain to blast insults and may prove important to establish correlates with observed localized brain-tissue changes.

## Data Availability

The datasets presented in this article can be made available upon written request to the corresponding author, along with a summary of the planned research and related analyses. Requests to access the datasets should be directed to jaques.reifman.civ@mail.mil.

## Author Disclaimer

The opinions or assertions contained herein are the private views of the authors and are not to be construed as official or reflecting the views of the United States (U.S.) Army, the Department of Defense (DoD), or The Henry M. Jackson Foundation for the Advancement of Military Medicine, Inc. (HJF). Any citations of commercial organizations and trade names in this report do not constitute an official U.S. Army, DoD, or HJF endorsement of approval of the products or services of these organizations. This paper has been approved for public release with unlimited distribution.
